# Binding of the angiogenic/senescence inducer CCN1/CYR61 to integrin α_6_β_1_ drives endocrine resistance in breast cancer cells

**DOI:** 10.18632/aging.203882

**Published:** 2022-02-11

**Authors:** Ingrid Espinoza, Lin Yang, Travis Vander Steen, Luciano Vellon, Elisabet Cuyàs, Sara Verdura, Lester Lau, Javier A. Menendez, Ruth Lupu

**Affiliations:** 1 Department of Laboratory Medicine and Pathology, Division of Experimental Pathology, Mayo Clinic, Rochester, 55905 MN, USA; 2 Stem Cells Laboratory, Institute of Biology and Experimental Medicine (IBYME-CONICET), Buenos Aires C1428ADN, Argentina; 3 Program Against Cancer Therapeutic Resistance (ProCURE), Metabolism and Cancer Group, Catalan Institute of Oncology, Girona 17005, Spain; 4 Girona Biomedical Research Institute, Salt, Girona 17190, Spain; 5 Department of Biochemistry and Molecular Genetics, College of Medicine, The University of Illinois at Chicago, Chicago, IL 60607, USA; 6 Department of Biochemistry and Molecular Biology Laboratory, Mayo Clinic Minnesota, Rochester, MN 55905, USA; 7 Mayo Clinic Cancer Center, Rochester, MN 55905, USA; 8 Current address: Department of Preventive Medicine, John D. Bower School of Population Health, University of Mississippi Medical Center, Jackson, MS 39216, USA; 9 Current address: Cancer Institute, School of Medicine, University of Mississippi Medical Center, Jackson, MS 39216, USA

**Keywords:** matricellular proteins, CYR61, integrins, estrogen receptor, tamoxifen

## Abstract

CCN1/CYR61 promotes angiogenesis, tumor growth and chemoresistance by binding to its integrin receptor α_v_β_3_ in endothelial and breast cancer (BC) cells. CCN1 controls also tissue regeneration by engaging its integrin receptor α_6_β_1_ to induce fibroblast senescence. Here, we explored if the ability of CCN1 to drive an endocrine resistance phenotype in estrogen receptor-positive BC cells relies on interactions with either α_v_β_3_ or α_6_β_1_. First, we took advantage of site-specific mutagenesis abolishing the CCN1 receptor-binding sites to α_v_β_3_ and α_6_β_1_ to determine the integrin partner responsible for CCN1-driven endocrine resistance. Second, we explored a putative nuclear role of CCN1 in regulating ERα-driven transcriptional responses. Retroviral forced expression of a CCN1 derivative with a single amino acid change (D125A) that abrogates binding to α_v_β_3_ partially phenocopied the endocrine resistance phenotype induced upon overexpression of wild-type (WT) CCN1. Forced expression of the CCN1 mutant TM, which abrogates all the T1, H1, and H2 binding sites to α_6_β_1_, failed to bypass the estrogen requirement for anchorage-independent growth or to promote resistance to tamoxifen. Wild-type CCN1 promoted estradiol-independent transcriptional activity of ERα and enhanced ERα agonist response to tamoxifen. The α_6_β_1_-binding-defective TM-CCN1 mutant lost the ERα co-activator-like behavior of WT-CCN1. Co-immunoprecipitation assays revealed a direct interaction between endogenous CCN1 and ERα, and *in vitro* approaches confirmed the ability of recombinant CCN1 to bind ERα. CCN1 signaling via α_6_β_1_, but not via α_v_β_3_, drives an endocrine resistance phenotype that involves a direct binding of CCN1 to ERα to regulate its transcriptional activity in ER+ BC cells.

## INTRODUCTION

**Figure 1:**
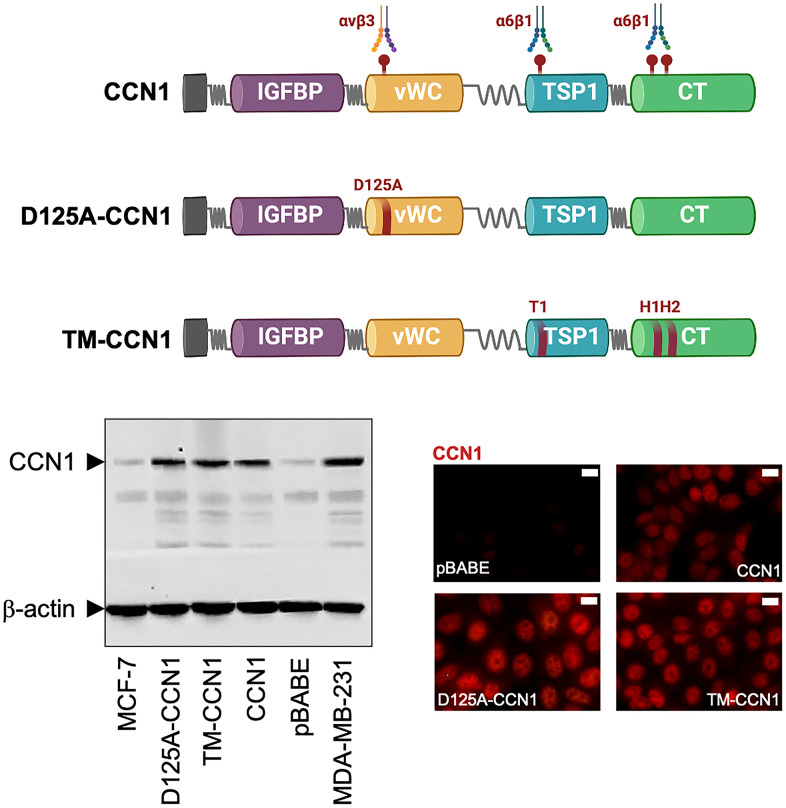
**Expression of CCN1 and CCN1 mutants in MCF-7 breast cancer cells.** Top: Schematic diagram showing the modular domain structure of wild-type CCN1 with the localization of several identified integrin-binding sites, and mutants either bearing the D125A mutation in vWC (D125A-CCN1) or combined mutations in T1, H1, and H2 in TSP1 and TC domains (TM-CCN1). *IGFBP*, insulin-like growth factor binding protein; *vWC*, von Willebrand factor type C repeats; *TSP-1*, thrombospondin type 1; *CT*, C-terminus. Bottom: Immunoblotting assessment of endogenous CCN1 protein in CCN1-overexpressing MDA-MB-231 cells and in MCF-7 cells retrovirally transduced with an empty vector (pBABE) or a vector containing either wild-type CCN1 or D125A-CCN1 and TM-CCN1 mutants. Microphotographs show representative *in situ* immunofluorescence staining of CCN1 in MCF-7/pBABE, MCF-7/CCN1, MCF-7/D125A-CCN1, and MCF-7/TM-CCN1 cells. Scale bar is 10 μm. Results are representative of three independent experiments.

CCN1 (also named cysteine-rich angiogenic inducer 61 [CYR61]) is an archetypal component of the CCN (CYR61, CTGF, NOV) family of matricellular proteins [1–4]. CCN1 has diverse developmental functions in early life (e.g., placental angiogenesis, vascular integrity, and cardiac morphogenesis) and also plays critical roles in inflammation, wound healing, and tissue repair in the adult [5–13]. Aberrantly expressed CCN1 correlates with numerous chronic inflammation-related diseases, including cancer [14–20]. The ability of CCN1 to interact directly with multiple binding partners, particularly cell-surface integrin receptors but also as-yet-unidentified proteins, underlies its functional versality [21, 22]. Crucially, the multifunctionality of CCN1 can be attributed to its multimodular architecture, in which the location of several receptor-binding sites throughout the modular domains of CCN1 physically links CCN1-triggered signaling events to biological activities in a cell- and context- (physiological versus pathological) dependent manner [reviewed in 23, 24]. For instance, the interaction of the V2 functional site at the von Willebrand factor type C repeat (vWC) domain of CCN1 with integrin α_v_β_3_ in endothelial and cancer cells is critical for angiogenic and proliferative activities in embryonic development and tumor growth. By contrast, the interaction of the T1, H1, and H2 functional sites at the carboxy-terminal (CT) domain with integrin α_6_β_1_ (T1) and heparan sulfate proteoglycans (H1, H2) in fibroblasts is critical for apoptosis and cellular senescence phenomena during fibrosis and wound healing [25–28] (Figure 1).

Our own previous studies and those of others have established a significant correlation between elevated levels of CCN1 and more advanced disease and metastatic phenotypes in *in vitro* breast cancer models and in patients [14–17, 20, 29–33]. Specifically, we demonstrated that the ability of CCN1 to drive breast tumor initiation, vascularization, and invasiveness, as well as to provide protection of breast cancer cells against chemotherapy-induced apoptosis, was largely mediated through binding to integrin α_v_β_3_, whose expression is also induced by CCN1 [30, 34, 35]. *In vitro* studies have also clarified the ability of CCN1 to overcome estrogen dependency and elicit resistance to the selective estrogen receptor (ER) modulators and down-regulators (SERMs/SERDs) tamoxifen and fulvestrant in ER-positive breast cancer cells [15–17, 29, 31]. Patients with CCN1-overexpressing, hormone-dependent breast cancer respond poorly to the aromatase inhibitor letrozole [32]. In this context, a recent study has highlighted a role for CCN1 in the development of endocrine resistance in patients with breast cancer [33], identifying it as a potential therapeutic target to overcome refractoriness to a wide-range of antiestrogen therapies. Nonetheless, it is not clear whether the ability of CCN1 to bypass estrogen-dependence and drive resistance to endocrine therapy relies on the interaction with its cell-surface integrin receptors (α_v_β_3_/α_v_β_5_ and α_6_β_1_) and/or with potential nuclear functions of CCN1 [36–38] that might affect ERα-driven transcriptional activity.

In the present study, we used previous mutational analyses showing that distinct integrin-binding sites of CCN1 can function independently of one another [25–28] to determine the signaling pathway through which CCN1 mediates endocrine resistance in breast cancer. We first employed site-specific CCN1 mutations specifically abolishing the receptor-binding sites to either α_v_β_3_/α_v_β_5_ or α_6_β_1_ to delineate the integrin partner responsible for CCN1-driven endocrine resistance in breast cancer. Also, given the intriguing possibility that the nuclear localization of CCN1 may regulate gene transcription, we explored a putative nuclear role for CCN1 in regulating ERα-driven transcriptional responses.

## RESULTS

### Generation of ER-positive breast cancer cells overexpressing CCN1 and α_v_β_3_/α_6_β_1_-binding-defective CCN1 mutants

Estrogen-dependent MCF-7 breast cancer cells, which naturally express very low levels of CCN1, were engineered to stably overexpress either wild-type CCN1 or the mutational derivatives D125A-CCN1, which exhibits a disrupted α_v_β_3_-binding site through the D125A mutation, and TM-CCN1, which abrogates all the T1, H1, and H2 binding sites to α_6_β_1_ (Figure 1) [25–28]. Immunoblotting procedures, which were performed following cell starvation to prevent the serum effect on CCN1 expression, confirmed that CCN1 was almost undetectable in MCF-7 parental and MCF-7/pBABE control cells but was noticeably elevated in MCF-7/CCN1, MCF-7/D125A-CCN1, and MCF-7/TM-CCN1 cells (Figure 1). CCN1 protein levels in MCF-7/CCN1, MCF-7/D125A-CCN1, and MCF-7/TM-CCN1 cells were comparable to those of MDA-MB-231 cells, a triple-negative breast cancer model naturally overexpressing CCN1 [15, 16]. Immunofluorescence analysis of CCN1 protein revealed that, in addition to its cytoplasmic location, CCN1 exhibited an apparent nuclear-staining pattern in the majority of MCF-7 breast cancer cell lines, with no evident differences between wild-type and mutant CCN1 (Figure 1).

### D125A-CCN1, but not TM-CCN1, phenocopies wild-type CCN1 to drive long-term acquisition of an estrogen-independent phenotype

**Figure 2:**
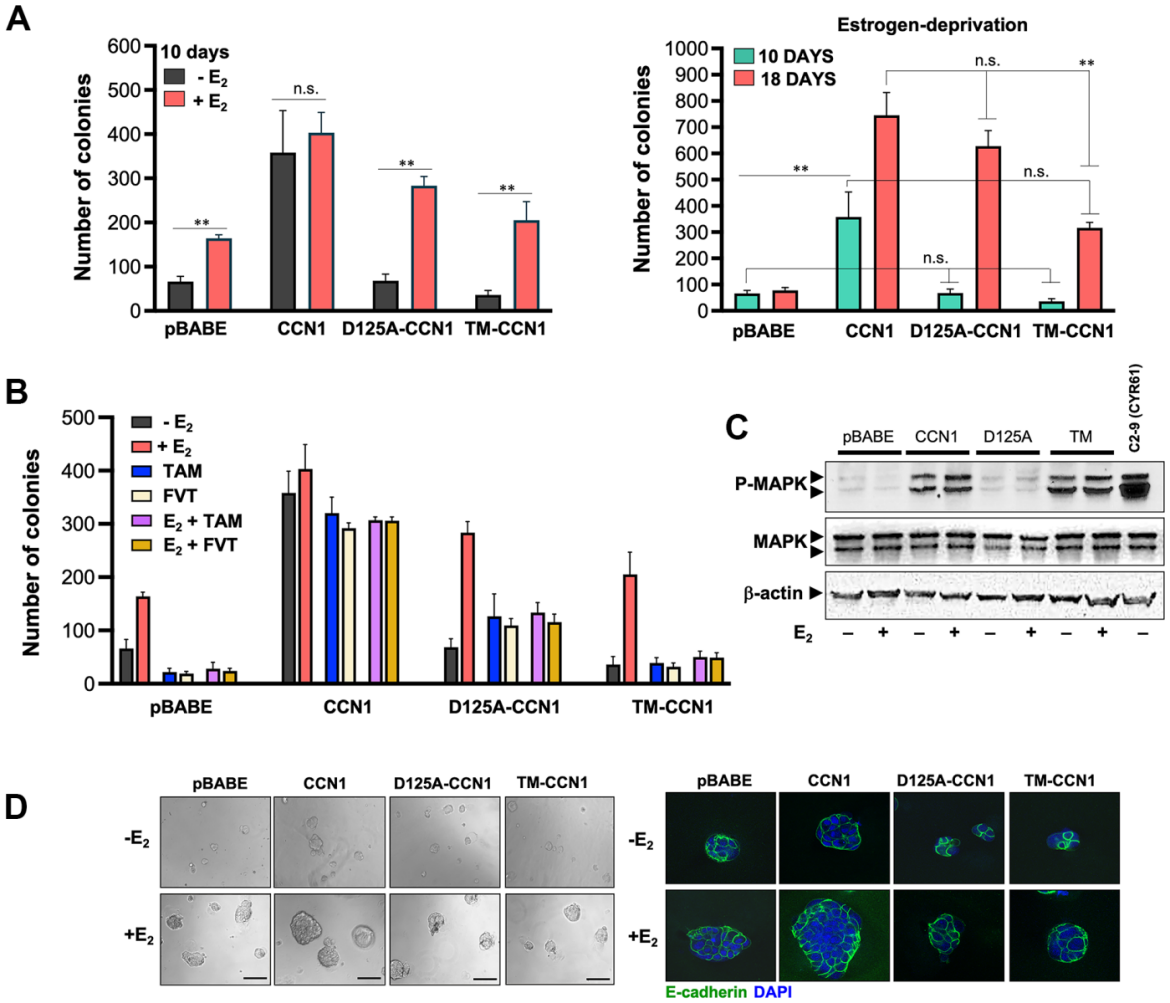
**CCN1 and D125A-CCN1, but not TM-CCN1, promote acquisition of an endocrine resistance phenotype in MCF-7 breast cancer cells.** (**A**, **B**) Estradiol (E_2_)-depleted cells were plated in soft agarose either containing or not E_2_ (10^−9^ M), tamoxifen (10^−7^ M), fulvestrant (10^−7^ M), their combinations, or vehicles only for either 10 or 18 days. Colony formation (≥50 μm) was assessed using a colony counter. Each experimental value represents the mean colony number (columns) ± S.D. (bars) from at least three separate experiments in which triplicate dishes were counted. (**C**) Immunoblot analyses of total and activated (phosphorylated) MAPK protein levels in MCF-7/pBABE, MCF-7/CCN1, MCF-7/D125A-CCN1, and MCF-7/TM-CCN1 cells. Blots were reprobed with an antibody for β-actin to control for protein loading and transfer. Results are representative of three independent experiments. (**D**) Phase contrast images of MCF-7/pBABE, MCF-7/CCN1, MCF-7/D125A-CCN1, and MCF-7/TM-CCN1 cells cultured in Matrigel^®^ in the absence or presence of E_2_ (10^−9^ M). Scale bar is 100 μm. 3D cultures were stained for E-cadherin and nuclei were counterstained with DAPI.

We assessed whether specific modification of CCN1-integrin(s) binding would impact the ability of CCN1 to modulate estrogen dependency of ER-positive breast cancer cells. To do this, we first compared short-term (10 days) anchorage-independent growth of MCF-7/pBABE, MCF-7/CCN1, MCF-7/D125A-CCN1, and MCF-7/TM-CCN1 cells by colony formation assays in soft agar (Figure 2A, left panel). Forced expression of wild-type CCN1 promoted robust anchorage-independent growth of MCF-7/CCN1 cells in the absence of estradiol supplementation. By contrast, neither MCF-7/D125A-CCN1 nor MCF-7/TM-CCN1 cells formed colonies in the absence of estradiol (Figure 2A, left panel). Addition of estradiol failed to increase further the already strong colony formation capacity of MCF-7/CCN1 cells. E_2_ supplementation augmented the anchorage-independent growth in MCF-7/D125A-CCN1 cells beyond that observed in E_2_-treated MCF-7/pBABE control cells (Figure 2A, left panel). Conversely, the estradiol-driven potentiation of anchorage-independent growth in MCF-7/TM-CCN1 cells was indistinguishable from that produced in MCF-7/pBABE control cells (Figure 2A, left panel).

We then re-assessed the patterns of anchorage-independent growth in the long-term absence of estrogens (up to 18 days in soft-agar). MCF-7/D125A-CCN1 cells were capable of strikingly circumvent estradiol requirement to form a similar number of colonies to those generated by MCF-7/CCN1 cells in the long-term (Figure 2A, right panel). The ability of D125A-CCN1 to drive long-term acquisition of an estrogen-independent phenotype was much less pronounced with the TM-CCN1 derivative. Thus, although capable of forming colonies after long term culture in the absence of estradiol, MCF-7/TM-CCN1 cells failed to fully recapitulate the highly-aggressive, estrogen-independent phenotype of MCF-7/CCN1 and MCF-7/D125A-CCN1 cells (Figure 2A, right panel).

### TM-CCN1, but not D125A-CCN1, loses the capacity of wild-type CCN1 to promote resistance to anti-estrogens

We next assessed whether modulation of CCN1 expression and/or specific modification of CCN1-integrin(s) binding would affect the anti-estrogen sensitivity of ER-positive breast cancer cells. Tamoxifen and fulvestrant completely inhibited the estradiol-stimulated anchorage-independent growth of MCF-7/pBABE cells, whereas forced expression of CCN1 fully abrogated the inhibitory effects of tamoxifen and fulvestrant on soft-agar colony formation irrespective of the presence or absence of estradiol (Figure 2B). Estradiol-independent colony formation capacity in MCF-7/D125A-CCN1 cells was weakly but significantly stimulated by tamoxifen and fulvestrant; such agonist effects on the anchorage-independent growth of MCF-7/D125A-CCN1 cells were not further enhanced by estradiol (Figure 2B). MCF-7/TM-CCN1 cells retained a *bona fide* endocrine-sensitive phenotype in which tamoxifen and fulvestrant failed to exhibit any agonist effect on the estradiol-independent colony formation capacity and estradiol stimulation failed to promote anchorage-independent growth in the presence of anti-estrogens (Figure 2B).

Because α_v_β_3_-dependent activation of the MAPK pathway was previously found to drive CCN1-directed cell survival and chemoresistance [30], we explored whether specific modulation of the CCN1-integrin(s) binding differentially altered ERK1/ERK2 activity in breast cancer cells. The activation status of MAPK was significantly higher in MCF-7/CCN1 cells than in matched control MCF-7/pBABE cells by immunoblotting analysis (Figure 2C). Abrogation of CCN1 binding to α_v_β_3_ fully prevented CCN1-driven MAPK hyperactivation in MCF-7/D125-CCN1 cells, but the abrogation of CCN1 binding to α_6_β_1_ fully retained the ability of wild-type CCN1 to activate MAPK in MCF-7/TM-CCN1 cells (Figure 2C).

### CCN1-driven endocrine resistance does not alter 3D breast cancer colony morphology

Because cell culture in three-dimensional (3D) extracellular matrix (ECM) is considered as a more relevant model system to evaluate cancer cell behavior [39, 40], we evaluated the size, form, and E-cadherin distribution of colonies formed by MCF-7/pBABE, MCF-7/CCN1, MCF-7/D125A-CCN1, and MCF-7/TM-CCN1 cells cultured in Matrigel^®^. MCF-7/CCN1 cells formed larger colonies than MCF-7/pBABE, MCF-7/D125A-CCN1, and MCF-7/TM-CCN1 cells, an effect that was more notable in the presence of estradiol (Figure 2D, left panels). Despite the obvious differences in their colony sizes when grown on top an ECM gel, the expression of E-cadherin was not down-regulated in none of the CCN1-overexpressing cell models (Figure 2D, right panels). Overexpression of wild-type CCN1 and abrogation of CCN1 binding to α_v_β_3_ in MCF-7/D125-CCN1 cells and to α_6_β_1_ in MCF-7/TM-CCN1 cells were insufficient to promote the formation of branching colonies in 3D Matrigel cultures – a hallmark of the invasive mesenchymal phenotype. Accordingly, MCF-7/pBABE, MCF-7/CCN1, MCF-7/D125A-CCN1, and MCF-7/TM-CCN1 cells all exhibited a mass-like morphology with disorganized nuclei and filled colony centers characteristic of luminal-like breast cancer cells [39, 40] (Figure 2D, right panels).

### CCN1 drives the constitutive activation of estrogen receptor transcriptional activity

**Figure 3:**
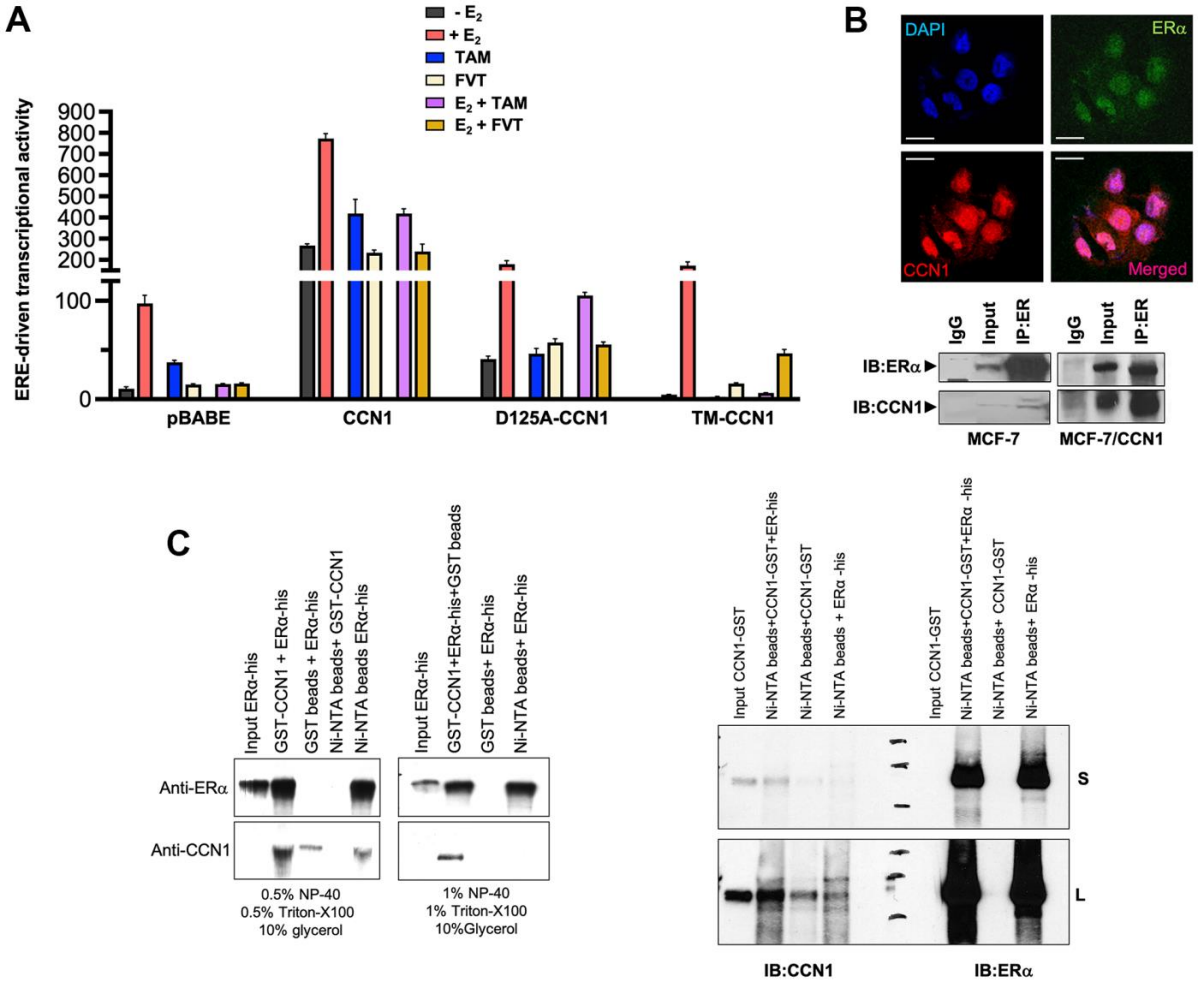
**CCN1 directly binds the estrogen receptor and regulates its transcriptional activity.** (**A**) MCF-7/pBABE, MCF-7/CCN1, MCF-7/D125A-CCN1, and MCF-7/TM-CCN1 cells were transiently with an ERE-Luciferase reporter (the ERE-containing reporter plasmid) and pRL/CMV (an internal reporter plasmid to control for transfection efficiency). Cells were incubated in the absence or presence of estradiol (E_2,_ 10^−9^ M), tamoxifen (10^−7^ M), fulvestrant (10^−7^ M), their combinations, or vehicles for 24 h, and cell extracts were analyzed for Luciferase activity. Data shown represent mean (columns) ± S.D. (bars) (*n=3*). (**B**) Top: Microphotographs show representative *in situ* immunofluorescence staining of CCN1 and/or estrogen receptor (ERα) in MCF-7/CCN1 cells. Scale bar is 10 μm. Bottom: ERα in the cell lysates of MCF-7 and MCF-7/CCN1 cells was immunoprecipitated and immunoblotted with anti-ERα and anti-CCN1 antibodies. (**C**) Representative immunoprecipitation results of His-tagged ERα and GST-CCN1 using immobilized Ni^2+^. Purified GST-CCN1 protein was incubated with human recombinant ERα-His protein and Ni-NTA His•Bind resin beads. As controls, ERα-His protein was incubated with GST-only beads or GST-CCN1 was incubated with Ni-NTA beads alone. Proteins retained in the beads were denatured and blotted with the indicated antibodies. Results in (**B**, **C**) are representative of three independent experiments. (S: Short exposure; L: Long exposure).

To evaluate the effects of CCN1 expression and/or specific modification of CCN1-integrin(s) binding on ERα-transactivation and estradiol responsiveness, we transfected MCF-7/pBABE, MCF-7/CCN1, MCF-7/D125A-CCN1, and MCF-7/TM-CCN1 cells together with a Luciferase reporter gene linked to the consensus Estrogen Response Element (ERE-Luciferase). Transfected cells were then evaluated for changes in the levels of basal (estradiol-independent) and induced (estradiol-stimulated) ERα activity in the absence or presence of anti-estrogens. MCF-7/CCN1 cells showed a very strong constitutive activation of ERα transcriptional activity in the absence of estradiol stimulation, which was largely reduced in MCF-7/D125A-CCN1 cells and fully prevented in MCF-7/TM-CCN1 cells (Figure 3A). Both tamoxifen and fulvestrant failed to suppress the constitutive hyperactivation of ERα-driven transcription in MCF-7/CCN1 cells irrespective of the presence or absence of estradiol. Fulvestrant, but not tamoxifen, suppressed estradiol-induced activation of ERE activity in MCF-7/D125A-CCN1 cells (Figure 3A). Similar to MCF-7/pBABE control cells, MCF-7/TM-CCN1 cells were exquisitely responsive to the ability of tamoxifen and fulvestrant to suppress estradiol-induced agonist transactivation of ERα transcriptional activity (Figure 3A).

### CCN1 directly interacts with the estrogen receptor

Given the nuclear staining pattern of CCN1 in MCF-7/CCN1 cells, we envisioned that CCN1 might interact with ERα. Double immunofluorescence staining of CCN1 and ERα suggested a nuclear co-localization of these proteins in MCF-7/CCN1 cells (Figure 3B, top panels). Co-immunoprecipitation assays of whole cell extracts using anti-ERα, anti-CCN1, and nonspecific IgG antibodies confirmed the interaction between endogenous CCN1 and ERα in MCF-7/CCN1 cells (Figure 3B, bottom panels). Such a strong CCN1-ERα interaction was not detected in immunoblot analyses of immunoprecipitates from CCN1-negative MCF-7/pBABE cells. *In vitro* approaches confirmed the specific ability of recombinant GST-CCN1 to bind recombinant poly-histidine-tagged ERα (Figure 3C).

## DISCUSSION

We show that CCN1/CYR61 signaling via α_6_β_1_, but not via α_v_β_3_/α_v_β_5_, drives an endocrine resistance phenotype that involves the unforeseen direct binding of CCN1 to ERα to regulate its transcriptional activity in breast cancer cells.

**Figure 4:**
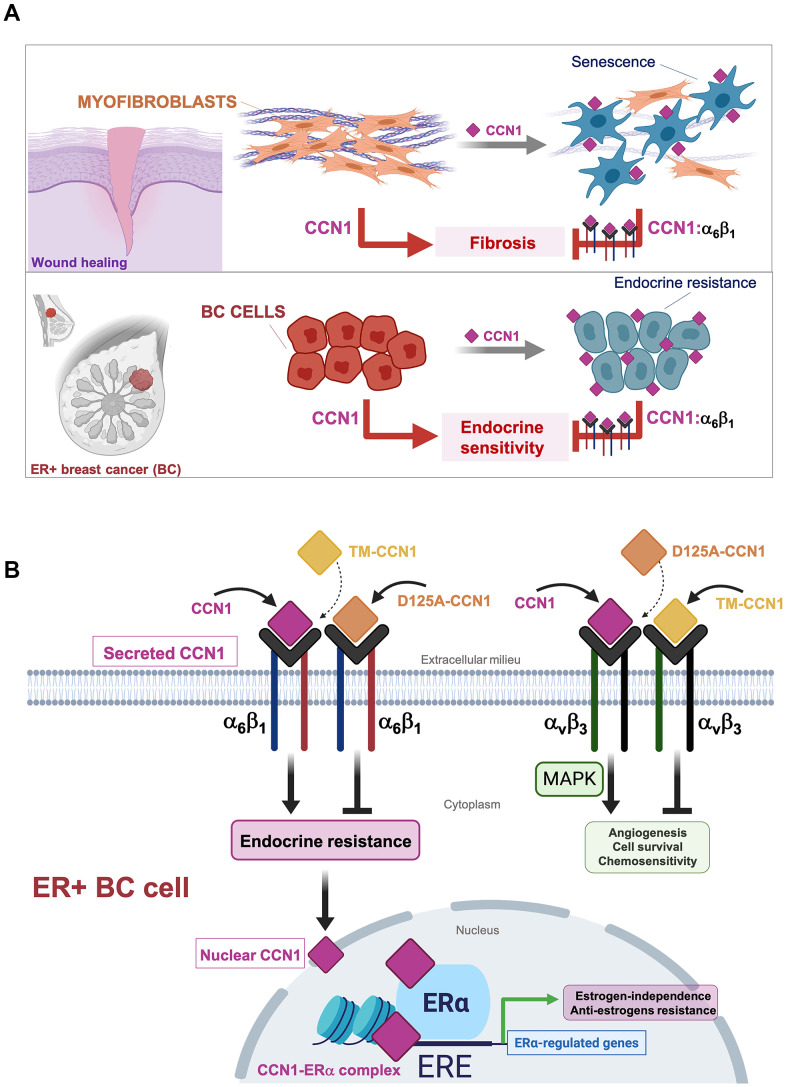
**Binding of the angiogenic/senescence inducer CCN1/CYR61 to integrin α_6_β_1_, but not to α_v_β_3_, drives endocrine resistance in breast cancer cells.** (**A**) Top: The binding of CCN1/CYR61 to its α_6_β_1_ receptor promotes myofibroblast senescence to impose self-limiting control on fibrogenesis during wound healing, thereby allowing tissue regeneration [11–13, 23, 48]. Bottom: CCN1 signaling via α_6_β_1_, but not via α_v_β_3_, drives an endocrine resistance phenotype in ER+ breast cancer cells. (**B**) The interaction between CCN1 and α_v_β_3_ is critical for angiogenic activities in endothelial cells and MAPK-related cell survival/chemosensitivity signaling in breast cancer cells. The interaction of CCN1 with α_6_β_1_ in fibroblasts is known to induce apoptosis or cellular senescence and has been widely regarded as a tumorigenesis-suppressing signaling mechanism. Here, we unveil the unforeseen capacity of CCN1 to signal through α_6_β_1_ in breast cancer cells to drive an endocrine resistant phenotype that might involve direct binding of CCN1 to ERα to regulate transcriptional events underlying estrogen-independence and anti-estrogen resistance in ERα-positive breast cancer cells. (ERE: Estrogen Response Elements).

Increased expression of CCN1 might promote angiogenesis, deregulated proliferation, enhanced cell survival and tumor invasiveness, and chemoresistance in breast cancer cells by activating integrin α_v_β_3_-driven cellular signaling [14, 17, 30, 41]. While accumulating evidence indicated that CCN1 serves a role in the development and maintenance of endocrine-resistant phenotypes in ER-positive breast carcinomas [16, 17, 29, 32, 33], it remained untested whether the anti-estrogen activities of CCN1 were similarly mediated through binding to integrin α_v_β_3_ and/or to another integrin receptor such as α_6_β_1_. To identify and dissect the differential functional roles of the CCN1-integrin interactions, we used a molecular strategy based on the specific disruption of integrin receptor-binding sites to test integrin-specific CCN1 functions in endocrine resistance. Importantly, CCN1 mutants employed in this study are biologically active, and their functional defects are indeed due to mutation of the specific receptor binding sites rather than structural perturbations [28]. Thus, whereas disruption of the CCN1 α_v_β_3_-binding site (D125A) specifically abolishes α_v_β_3_- but not α_6_β_1_-driven functions, a triple-CCN1 mutant disrupting all the α_6_β_1_-binding sites (TM-CCN1) specifically abolishes α_6_β_1_-dependent functions without affecting any of the α_v_β_3_-mediated activities of CCN1 [23, 28]. We show that a single amino acid mutation in the α_v_β_3_ binding site within a 20-amino acid sequence (V2) in CCN1 failed to suppress the endocrine resistance phenotype induced by overexpressing the wild-type form of CCN1 in ER-positive MCF-7 breast cancer cells. Because D125A does not impair the binding of CCN1 to α_6_β_1_, but fully prevents α_v_β_3_-mediated intracellular signaling including induction of MAPK––which was previously demonstrated to drive chemoresistance in breast cancer cells [30]–CCN1-driven activation of MAPK appears to be dispensable for CCN1-driven endocrine resistance in breast cancer. Conversely, the CCN1 mutant TM, which abrogates all the T1, H1, and H2 binding sites to α_6_β_1_ but maintains its capacity to bind α_v_β_3_ (enabling the sustained activation of MAPK), fails to bypass the estrogen requirement for anchorage-independent growth or to promote resistance to tamoxifen. To our knowledge, this is first demonstration that the interaction between CCN1 with α_6_β_1_, which is known to induce apoptosis or cellular senescence in fibroblasts to regulate the inflammatory response and control fibrosis during wound healing [11–13, 23], can be co-opted by ER-positive breast cancer cells to over-ride estrogen dependency and evade the growth-inhibitory effects of anti-estrogens (Figure 4A). Stimulation of breast cancer cell proliferation by estrogen and ERα might be, in part, due to the inhibition of senescence-like growth induced by oncogenic events in ER-positive breast cancer cells [42]. Suppressing ERα signaling with anti-estrogens such as tamoxifen is known to induce senescence-like phenotypes via induction of reactive oxygen species (ROS) [43]. Future studies are required to clarify whether the conversion of CCN1-overexpressing ER-positive breast cancer cells to an antiestrogen-resistant phenotype might associate with a shift toward a pro-oxidant environment as a result of the robust augmentation of ROS levels through binding of CCN1 to integrin α_6_β_1_ [44–46].

Despite the absence of a classical nuclear localization signal, CCN1 has unexpectedly been detected in the nucleus of cells [36]. Earlier studies suggested the intriguing possibility that CCN1 might regulate nuclear gene transcription through direct binding to DNA and/or to DNA-binding proteins [23, 36–38]. Here we found that overexpression of wild-type CCN1 promoted estradiol-independent transcriptional activity of ERα and enhanced ERα agonist response to tamoxifen. Moreover, we identified CCN1 as a previously unrecognized ERα-interacting protein and co-localizing with ERα in cell nuclei. Because the balance of coactivator and corepressor proteins in a cell may determine the response of the ERα to a particular ligand, these findings, overall, appear to illuminate an unforeseen coactivator-like behavior of nuclear CCN1 that could reduce the antagonist activity of tamoxifen-bound ERα. Intriguingly, whereas secreted wild-type CCN1 significantly modified ERα transcriptional activity, the tested secreted CCN1 mutants that failed to interact with either α_v_β_3_ or α_6_β_1_ notably differed in their ability to alter ERα-driven gene transcription (Figure 4B). The α_v_β_3_-binding-defective D125A-CCN1 mutant was less potent than wild-type CCN1 at promoting estradiol-independent ERα transcriptional activity, but still retained its capacity to promote an ERα agonist response to tamoxifen in the presence of estradiol. It cannot, therefore, be excluded that bi-directional cross-talk between integrin α_v_β_3_ and ERα via ERK1/ERK2 activation in membrane-associated and/or cytosol localizations, which may result in the phosphorylation of nuclear tamoxifen-liganded ERα and its associated coactivators, might be part of the CCN1-driven endocrine resistant phenotype in breast cancer cells. It is noteworthy that connective tissue growth factor (CTGF), another archetypal member of the CCN family of matricellular proteins, has been shown to physically and functionally associate with ERα to inhibit its transcriptional activity as well as the expression of estradiol-responsive genes [47]. The interaction between the CTGF thrombospondin type I repeat, a cell attachment motif, and the DNA-binding domain of ERα was required for the repression of estrogen-responsive transcription by CTGF [47]. Here we show that the α_6_β_1_-binding-defective TM-CCN1 protein entirely lacked the ability of wild-type CCN1 to exhibit an ERα co-activator-like behavior. While these data might suggest that binding to α_6_β_1_ is largely responsible for the capacity of CCN1 to regulate ERα transcriptional activity in an endocrine-resistant phenotype, future studies will be needed to clarify whether a direct interaction between CCN1 and ERα, which might be disrupted in the case of TM-CCN1, is required for the activation of estrogen/tamoxifen-responsive transcription by CCN1 in endocrine-resistant breast cancer cells (Figure 4).

CCN1/CYR61 might promote enhanced angiogenesis and deregulated proliferation and chemoresistance in breast tumors by binding to and activating α_v_β_3_ integrin signaling. CCN1/CYR61 promotes resolution of tissue fibrosis through induction of cellular senescence in myofibroblasts by engaging integrin α_6_β_1_ [11–13, 23]. Our present description of a novel role for the angiogenic/senescence inducer CCN1 in driving antiestrogen-resistance via α_6_β_1_ might provide a starting point to accelerate the development of CCN1/α_6_β_1_ integrin antagonists to therapeutically prevent the emergence of endocrine resistant phenotypes in ER-positive breast carcinomas.

## MATERIALS AND METHODS

### Cell culture

MCF-7 breast cancer cells were obtained from the American Type Culture Collection (ATCC) and were grown in phenol red-containing improved MEM (IMEM, Biosource International, Camarillo, CA, USA) supplemented with 5% fetal bovine serum (FBS) and 2 mM L-glutamine at 37° C in a humidified atmosphere of 95% air and 5% CO_2_. MCF-7 cells were authenticated to ensure their identity using a short tandem repeat profiling method provided by the Genotyping Shared Resource at Mayo Clinic Rochester.

MCF-7 cells were engineered to overexpress either wild-type CCN1 or CCN1 mutants with a single amino acid change (D125 to A), which abrogates binding to α_v_β_3_/α_v_β_5_-dependent activities (CCN1-D125A) or CCN1 disrupted in all three T1, H1, and H2 sites that completely abolishes α_6_β_1_-mediated activities (CCN1-TM), which were generated as described [25–28]. For virus production, TSA54 cells were grown in 10-cm dishes until 70–80% confluency and were transfected using FuGENE 6 (Roche Biochemicals, Indianapolis, IN, USA) with pBABE-Puro Retroviral Vector (10 μg) or CCN1 mutants cloned into the pBABE-Puro vector (10 μg) plus packaging plasmid PIK (10 μg). Twenty-four hours after transfection, the medium containing viral particles was collected and filtered through a 0.45-μm filter. MCF-7 cells growing at 70–80% confluency were infected twice with the viral particles and selected with 2.5 μg/ml puromycin. Stable transfected pools were maintained in puromycin-containing media for 4 weeks. CCN1/CYR61 expression levels were monitored by immunoblotting and immunofluorescence. Cells were regularly tested to confirm the absence of mycoplasma using the MycoAlert^®^ Mycoplasma Detection Kit (Lonza, Walkersville, MD, USA).

### Immunoblotting analysis of CCN1

Cells were serum-starved overnight, washed twice with phosphate buffered saline (PBS) and lysed in a buffer (20 mM Tris, pH 7.5, 150 mM NaCl, 1 mM EDTA, 1 mM EGTA, 1% Triton X-100, 2.5 mM sodium pyrophosphate, 1 mM β-glycerolphosphate, 1 mM Na_3_VO_4_, 1 μg/mL leupeptin, 1 mM phenylmethylsulfonylfluoride) for 30 min on ice. Lysates were cleared by centrifugation (15 min at 14,000 rpm at 4° C). Protein content was determined against a standardized control using the Pierce Protein Assay Kit (Rockford, IL, USA). Equal amounts of protein (50 μg) were resuspended in 5× Laemmli sample buffer and denatured for 5 min at 99° C. Proteins were resolved by electrophoresis in 10% SDS-PAGE gels, and transferred to PVDF membranes (Amersham Biosciences Ltd., Little Chalfont, Bucks, UK). Non-specific binding was minimized by blocking membranes with PBS-T (PBS and 0.5% Tween 20) containing 5% (w/v) non-fat dry milk for 1 h at room temperature. Membranes were washed in PBS-T and incubated overnight with a 1:2000 dilution of a rabbit anti-CCN1 polyclonal antibody (ab2026, Novus Biologicals, Inc., Littleton, CO, USA) at 4° C. After three washes with PBS-T, blots were incubated with 1:2000 dilution of a horseradish peroxidase-linked donkey anti-rabbit IgG secondary antibody for 45 min, and immunoreactive bands of CCN1 were detected using the enhanced chemiluminescence reagent (Pierce). Blots were re-probed with an antibody for β-actin goat polyclonal antibody (Santa Cruz Biotechnology, Santa Cruz, CA, USA). Densitometric values of CCN1 protein bands were quantified using the Scion Imaging software (Scion Corp., Frederick, MD, USA).

### *In situ* immunofluorescence staining

Cells were seeded at a density of 5×10^3^ cells/well in an 8-well chamber slide (Nalge Nunc International, Rochester, NY, USA). After 24 h of incubation, cells were washed with PBS, fixed in 4% paraformaldehyde in PBS for 15 min at room temperature, permeabilized with 0.2% Triton X-100/PBS for 15 min, and stored overnight at 4° C with 10% horse serum in PBS. Cells were then washed and then incubated for 1 h with an anti-CCN1 antibody diluted 1:200 in 5% BSA. After extensive washes, the cells were incubated for 1 h with a TRITC-conjugated anti-rabbit IgG secondary antibody (Jackson ImmunoResearch Labs, West Grove, PA, USA) diluted 1:200 in 5% BSA. The cells were washed five times with PBS and mounted with VECTASHIELD+DAPI (Vector Laboratories, Burlingame, CA, USA). As controls, cells were stained with primary or secondary antibody alone. No significant fluorescence was found in control experiments (data not shown). Indirect immunofluorescence was recorded on a Zeiss microscope (Jena, Germany). Images were noise-filtered, corrected for background, and prepared using Adobe Photoshop (San Jose, CA, USA).

### Soft agar colony formation assays

The efficiency of colony formation in liquid culture was determined by monitoring anchorage-independent cell growth in soft-agar. Cells were grown in phenol red-free IMEM and 5% charcoal calf serum (CCS) for 5 days in T-75 flasks to deplete estrogen. A bottom layer of 1.5 mL (2×) phenol red-free IMEM containing 1.2% agar and 10% CCS was prepared in 6 well plates. After the bottom layer solidified, cells (20,000 cells/well) were added in a 1 mL top layer containing either estradiol (10^-9^ M) and/or the anti-estrogens 4-OH-tamoxifen (10^-7^ M) or fulvestrant (10^-7^ M) in 0.7% agar and 10% CCS. Plates were incubated in a humidified 5% CO_2_ incubator at 37° C, and colonies measuring ≥50 μm were counted after 10–18 days using a cell colony counter (Optronix GelCount™, Abingdon, UK) after staining with nitroblue tetrazolium (Sigma-Aldrich, St. Louis, MO, USA). Assays were carried out in triplicate.

### Three-dimensional culture on Matrigel^®^

Single-cell suspensions of cells were prepared using trypsin. Cells (2×10^3^/well) in 0.4 mL of 2% Matrigel^®^ in 1× IMEM were then plated on top of a polymerized layer of 100% Matrigel^®^ using 8-well chamber slides. Cells were treated with estradiol and medium was replenished every 3 days. Control wells were maintained in medium containing 5% FBS. Cultures were kept for 5 days. Phase-contrast images were obtained under ×100 magnification.

### ERE-Luciferase activity

ER transcriptional activity was assessed using an ERE-driven reporter assay. Cells were propagated in estradiol-deprived (phenol red-free) IMEM supplemented with 5% CCS for 4 days before the onset of experiments, thereby ensuring the complete depletion of estradiol-like compounds from the medium. For experiments, cells were seeded into 12-well plates at 1×10^5^ cells/well. Cells were transfected using FuGENE 6 (Roche Biochemicals) with 0.75 μg/well of the estrogen-responsive reporter (ERE), containing a *Xenopus* vitellogenin A_2_-derived ERE, along with 0.05 μg/well of the internal control plasmid pRL-CMV, employed to correct for transfection efficiency. After 18 h, the transfected cells were washed and then incubated in fresh medium containing 5% CCS, supplemented with estradiol (10^-9^ M), tamoxifen (10^-7^ M), fulvestrant (10^-7^ M), or their combinations, as specified. Approximately 24 h after treatments, Luciferase activity from cell extracts was measured using a Dual Luciferase Assay System (Promega, Madison, WI, USA) on a TD-20/20 luminometer (Turner Designs, Sunnyvale, CA, USA). The magnitude of activation in ERE-Luciferase-transfected cells treated with the vehicle was determined after normalization to the activity of pRL-CMV and was defined as 1.0-fold. This control value was used to calculate the relative (fold) change in transcriptional activities of ERE-Luciferase-transfected cells in response to treatments after normalization to pRL-CMV activity. All data were normalized as the ratio of raw light units to pRL-CMV unit corrected for pRL-CMV activity, and were shown as the mean **±** SD from three separate experiments performed in triplicate.

### *In vitro* binding assays

The CCN1 coding sequence was cloned in-frame with the glutathione S-transferase (GST) gene at the EcoRI and SalI sites in the pGEX-4T1 vector. Production and purification of the CCN1-GST fusion protein was carried out as described [49]. Recombinant human estrogen receptor alpha (His-tag) (ab240853) was purchased from Abcam (Cambridge, UK). Ni-NTA His•Bind Resin, a high-performance Ni^2+^-charged agarose used for rapid one-step purification of proteins containing a His•Tag sequence by metal chelation chromatography, was purchased from Sigma-Aldrich (70666-3). Pierce™ Glutathione Agarose was obtained from ThermoFisher Scientific (San Jose, CA, USA) (16102BID). GST-CCN1 fusion protein was incubated with recombinant human estrogen receptor His-tagged protein and Ni-NTA His beads in the incubation buffer (pH 7.35, 150 mM NaCl, 0.5 mM EDTA, 50 mM Tri-HCl and 0.5% NP-40) at 4° C for 4 hours. After the incubation, the beads were washed for 15 min with washing buffer (pH 7.35, 0.5–1% NP-40+0.5–1% Triton-X100+10% glycerol) on a shaker in the cold room 5 times. The protein complexes bound to the beads were separated with SDS-PAGE and blotted with the indicated antibodies.

### Statistical analysis

For all experiments, at least 3 independent experiments were performed with n≥3 replicate samples per experiment. Data were presented as mean ± S.D. Comparisons of means of ≥3 groups were performed by one-way analysis of variance and Dunnett’s t-test for multiple comparisons using GraphPad Prism (GraphPad Software, San Diego, CA, USA). In all studies, p-values <0.05 and <0.005 were considered to be statistically significant (denoted as * and **, respectively). All statistical tests were two-sided.
